# Experimental characterization of two archaeal inosine 5'-monophosphate cyclohydrolases

**DOI:** 10.1371/journal.pone.0223983

**Published:** 2019-10-17

**Authors:** Caroline A. Hunter, Nicholas I. Plymale, Kevin M. Smee, Catherine A. Sarisky

**Affiliations:** Department of Chemistry, Roanoke College, Salem, Virginia, United States of America; Consejo Superior de Investigaciones Cientificas, SPAIN

## Abstract

There is variability as to how archaea catalyze the final step of *de novo* purine biosynthesis to form inosine 5’-monophosphate (IMP) from 5-formamidoimidazole-4-carboxamide ribonucleotide (FAICAR). Although non-archaea almost uniformly use the bifunctional PurH protein, which has an N-terminal IMP cyclohydrolase (PurH2) fused to a C-terminal folate-dependent aminoimidazole-4-carboxamide ribonucleotide (AICAR) formyltransferase (PurH1) domain, a survey of the genomes of archaea reveals use of PurH2 (with or without fusion to PurH1), the “euryarchaeal signature protein” PurO, or an unidentified crenarchaeal IMP cyclohydrolase. In this report, we present the cloning and functional characterization of two representatives of the known IMP cyclohydrolase families. The locus TK0430 in *Thermococcus kodakarensis* encodes a PurO-type IMP cyclohydrolase with demonstrated activity despite its position in a cluster of apparently redundant biosynthetic genes, the first functional characterization of a PurO from a non-methanogen. Kinetic characterization reveals a K_m_ for FAICAR of 1.56 ± 0.39 μM and a k_cat_ of 0.48 ± 0.04 s^-1^. The locus AF1811 from *Archaeoglobus fulgidus* encodes a PurH2-type IMP cyclohydrolase. This *Archaeoglobus fulgidus* PurH2 has a K_m_ of 7.8 ± 1.8 μM and k_cat_ of 1.32 ± 0.14 s^-1^, representing the first characterization of an archaeal PurH2 and the first characterization of PurH2 that naturally occurs unfused to an AICAR formyltransferase domain. Each of these two characterized IMP cyclohydrolases converts FAICAR to IMP *in vitro*, and each cloned gene allows the growth on purine-deficient media of an *E*. *coli* purine auxotroph lacking the *purH2* gene.

## Introduction

**Fig 1:**
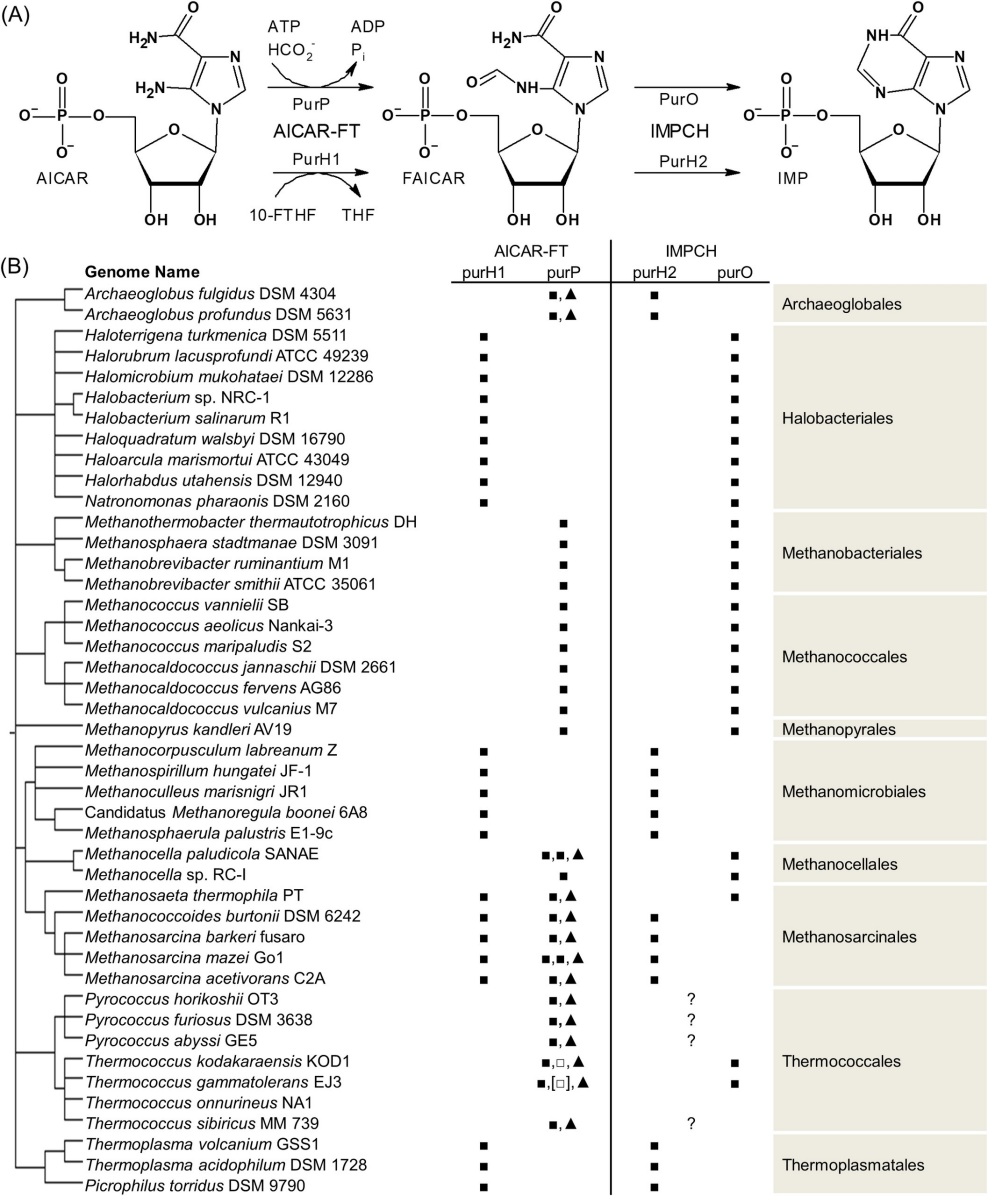
Reactions studied and phylogenetic patterns. (A) The enzymatic conversion of AICAR to FAICAR and FAICAR to IMP. Non-archaea use PurH for both steps, while archaea are more variable. Abbreviations used: AICAR-FT, AICAR formyltransferase. 10-FTHF, 10-formyltetrahydrofolate. IMPCH, IMP cyclohydrolase. Other abbreviations are as used in the text. (B) Presence of genes encoding putative AICAR formyltransferases and IMP cyclohydrolases in selected euryarchaea, adapted from [1]. Symbols used: ■ denotes a gene that is a good match. □ denotes a match with some problems, as described in more detail in [1]. ▲ is used to represent a cluster II PurP protein. [■] indicates that the expected gene is split into two adjacent loci. Where a "?" appears, an IMP cyclohydrolase gene is necessary for an otherwise complete purine biosynthesis pathway to be functional, but no gene candidate has been identified.

A *de novo* purine biosynthesis pathway is found in complete form in the genomes of all but a few free-living organisms. In most Bacteria and Eukarya, the final two steps from 5'-phosphoribosyl-5-amino-4-imidazolecarboxamide (AICAR, CID 65110) to inosine 5’-monophosphate (IMP, CID 135398640) in the *de novo* pathway are catalyzed by the bifunctional PurH protein, containing a C-terminal 10-formyltetrahydrofolate-dependent AICAR formyltransferase domain (“PurH1”) that produces 5'-phosphoribosyl-5-formamido-4-imidazolecarboxamide (FAICAR, CID 166760) and an N-terminal IMP cyclohydrolase domain (“PurH2”) that converts FAICAR to IMP. In Archaea, the enzymes involved are more diverse, with a patchy distribution of two unrelated IMP cyclohydrolases (PurO and PurH2) and two AICAR formyltransferases (10-formyltetrahydrofolate-requiring PurH1 and folate-independent PurP). There is little correlation between the presence of genes encoding 10-formyltetrahydrofolate-requiring PurH1 or formate-requiring PurP and the presence of genes encoding PurO or PurH2, with all four possible patterns occurring in archaea, as previously described [1, 2] and shown in Fig 1. In this paper, we report the kinetic characterization of two selected archaeal IMP cyclohydrolases, one each of the PurO and PurH2 types, to increase the diversity of experimentally-characterized IMP cyclohydrolases within the archaeal domain. This work represents the first characterization of a PurO-type IMP cyclohydrolase from a non-methanogen and the first characterization of an archaeal PurH2-type protein.

Although they catalyze the same reaction, there is no structural or sequence similarity between the two known families of IMP cyclohydrolases. The PurO IMP cyclohydrolase is a NTN-hydrolase fold, but lacks the active site nucleophile and autocatalytic self-processing common to other members of the superfamily [3]. Three sidechains (arginine, glutamate, and tyrosine) are postulated to catalyze the reaction by stabilizing an iminol tautomer of FAICAR and activating the formimino group to nucleophilic attack [3]. In contrast, the PurH2 domain (usually present as part of full-length PurH) is related to methylglyoxal synthase, with the important contacts to the new purine ring coming from the protein backbone, and primarily functioning to orient the FAICAR for cyclization [4].

The PurO from *Methanocaldococcus jannaschii*, encoded by locus MJ0626, was first identified by purification from whole cell extracts followed by cloning and heterologous over-expression of the protein identified from the cell extracts [5]. The closely-related PurO from *Methanothermobrevibacter thermoautotrophicus*, encoded by MTH1020, has since been over-expressed and structurally characterized [3]. Inspection of the genomes of fully-sequenced archaea reveals that genes encoding PurO are largely limited to a subset of methanogens and the Halobacteria. Based on the limited sequence data available at the time, PurO was originally described as an archaeal signature protein [5], but it is uniformly absent from the Crenarchaeaota and Thaumarchaeota. It might be described as a Euryarchaeal signature protein (although present in only 34 of 95 sequenced Euryarchaea [2]), except that genes encoding proteins similar to PurO are also reported in a tiny fraction of sequenced bacteria (22 of 1266 [2]).

A gene encoding an apparent PurO-type IMP cyclohydrolase is present in *Thermococcus kodakarensis*, *Thermococcus gammatolerans*, *Palaeococcus ferrophilus*, and *Thermococcus sp*. *PK*, but absent from some other Thermococci and the closely related Pyrococci. Where *purO* occurs in the Thermococci, it appears to form part of an “alien operon”, a region of the genome that has been recently transferred [6]. Several genes in the region of the *Thermococcus kodakarensis purO-*like locus (TK0430) do not match the rest of the genome for codon usage [6]. Additionally, several genes in the vicinity of these *purO* loci appear to be redundant and possibly non-functional, with a more conserved version of the predicted protein found encoded elsewhere on the genome [1]. The genomes of *Thermococcus kodakarensis*, *Thermococcus gammatolerans*, *Palaeococcus ferrophilus*, and *Thermococcus sp*. *PK* all have a predicted gene cluster containing hydroxymethylpyrimidine synthase (*thiC*), a duplicate and divergent *purC*, the unexpected *purO*, and a divergent duplicate *purP*-like gene, in that order. There is also genomic evidence for a history of transposase/integrase activity in the vicinity of this cluster, which may suggest how the ancestor of these organisms came to acquire this gene cluster. While this predicted protein has the residues determined to be important for binding and catalysis in the two PurOs characterized in methanogens [3], overall amino acid identity between *Thermococcus kodakarensis* PurO and previously-characterized PurOs is only 32–35%. The genomes of closely-related Pyrococci have no evidence for an IMP cyclohydrolase of either the PurO or PurH2 type, despite an otherwise complete predicted pathway, implying the existence of a third IMP cyclohydrolase enzyme, previously posited [1, 2, 7]. Thus, given this confusing genomic context modest sequence identity to experimentally-characterized PurOs, the predicted PurO from *T*. *kodakarensis* merited functional characterization. Cloning of the TK0430 locus followed by kinetic characterization of heterologously-expressed protein and heterologous complementation assays were undertaken to determine whether this gene encoded an active IMP cyclohydrolase.

PurH2 most commonly occurs in non-archaea as full-length PurH, with the IMP cyclohydrolase domain at the N-terminus and the AICAR formyltransferases domain at the C-terminus. Studies on human PurH have shown that there is no channeling between the AICAR formyltransferase domain (PurH1) and the IMP cyclohydrolase domain (PurH2) [8]. Although they are nearly always fused in the non-archaea, the human PurH1 and PurH2 domains are functional when individually produced [9], as are those of *Staphylococcus lugadensis* [10]. Despite a pronounced trend towards production of full-length PurH in non-archaea, examination of the Archaea revealed predicted PurH2s without fusion to PurH1 in the Archaeoglobi [1]. The *Archaeoglobus fulgidus* locus AF1811 encodes a protein with only 25% amino acid identity to the characterized human protein [11], and only 29% identity to the characterized *Staphylococcus lugadensis* protein [10]. In this paper, we report the first characterization of the gene product of *Archaeoglobus fulgidus* AF1811, predicted to be an unfused PurH2 domain, which is also the first characterization of any sort of PurH from the domain Archaea.

## Materials and methods

### Plasmid construction

Directional cloning was performed using standard methods [12]. Primers for PCR were designed to amplify the TK0430 locus from *T*. *kodakaraensis* genomic DNA (kind gift of H. Atomi) and AF1811 from *A*. *fulgidus* genomic DNA (American Type Culture Collection, Manassas, VA). PCR products were purified, digested with appropriate restriction enzymes, and ligated into doubly-digested and antarctic phosphatase-treated pMAL-c5e vector (New England Biolabs, Ipswich, MA), producing an in-frame fusion with the maltose binding protein (MBP), as detailed in S1 File. An IPTG-inducible T5-lac promoter preceded the fusion construct. Ligation products were transformed into XL1-Blue cells and plated on LB plates with 100ug/mL ampicillin. Selected colonies were grown overnight in liquid medium, followed by plasmid isolation. The presence and orientation of inserts were verified by sequencing using the malE-rev and malE-for primers (Elim Biopharmaceuticals, Hayward, CA). pPurH1 was constructed from the ASKA plasmid purH (from National BioResource Project, JAPAN), using site directed mutagenesis to produce a 92 amino acid deletion near the N-terminus, as described in S1 File.

### Protein production

TK0430 and AF1811 were over-expressed in Rosetta2 (DE3) cells (EMD Biosciences, San Diego, CA). Cells were grown in LB with antibiotic selection (34μg/mL chloramphenicol and 100μg/mL ampicillin), followed by induction with 1mM IPTG. Induction proceeded overnight at room temperature. Cells were pelleted, resuspended in buffer containing 0.3M TES/K^+^ (potassium N-tris(hydroxymethyl)methyl-2-ammonioethanesulfonate, pH 7.3) and 10mM Mg^2+^, and disrupted by sonication. After pelleting of cell debris, TK0430 and AF1811 proteins were isolated from the soluble protein fraction by binding to amylose resin (New England Biolabs) equilibrated with buffer (1mM dithiothreitol (DTT), 200mM sodium chloride (NaCl), 1mM ethylenediaminetetraacetic acid (EDTA), 20mM Tris-HCl). After washing the resin with the same buffer, the target protein was eluted with buffer supplemented with 0.5M maltose. Buffer was exchanged by size exclusion chromatography using Sephadex G-25 medium resin with protein elution in 0.05M Tris, pH 7.3. Purification was monitored by SDS-polyacrylamide gel. Purified proteins were quantitated by UV absorbance at 280nm.

### FAICAR synthesis

FAICAR was chemically produced using the method of Mueller and Benkovic [13], with the following modifications: the reaction was performed at 1/10^th^ the published scale; the AICAR starting material (EMD Biosciences) was the free acid instead of the Ba^2+^ salt; the acetic anhydride and formic acid were pre-incubated at 37°C for one hour prior to addition to the AICAR; the reaction was allowed to proceed for 1–2 hours at 37°C followed by another 8–16 hours at room temperature; a vacuum concentrator was used in place of lyophilization. Although previous authors reported near stoichiometric conversions [5, 13], several synthesis attempts yielded no better than 70% yield (as assessed by HPLC, below), with unreacted starting material and side products (suspected to be the result of acetylation or off-site formylation) present.

After pH adjustment to neutral pH with ammonia, FAICAR was purified from unreacted starting materials and side products on a Varian ProStar HPLC with a Phenomenex Luna Omega PS C18 (250 x 4.6 mm) HPLC column, with 5mM tetrabutylammonium phosphate and 60mM ammonium phosphate (95% aqueous, 5% methanol) as the mobile phase at pH 6.0. Analytes were monitored by absorbance at 260nm. Solvent was removed overnight with a vacuum concentrator, followed by a second HPLC purification using 100% aqueous triethylammonium acetate at pH 6.0, followed by solvent and buffer removal by on a vacuum concentrator. Purity of >95% was confirmed by reinjection of purified material on the HPLC, using the tetrabutylammonium phosphate-containing conditions.

### Product and substrate identification

To identify the product formed, 75μM FAICAR in 0.050M Tris buffer at pH 7.3 was incubated with purified enzyme (0.22 μM TK0430 or 0.28 μM AF1811) or a buffer blank at 60°C for 30 minutes, followed by HPLC on a Phenomenex Luna Omega PS C18 column (250 x 4.6 mm) with 5mM tetrabutylammonium phosphate and 60mM ammonium phosphate (95% aqueous, 5% methanol) as the mobile phase at pH 6.0, with a flow rate of 1 mL/min. Monitoring of absorbance was a 248nm. Retention times were compared to authentic IMP (75 μM in 0.050M Tris buffer at pH 7.3).

### Kinetics studies

Kinetics studies were performed in 0.050M Tris, pH 7.3. Reactions were run at 50°C, with absorbance monitored at 248nm on a Shimadzu UV-2401 PC. FAICAR stock solutions were quantitated by absorbance at 248nm on a Nanodrop 2000c using the previously-reported extinction coefficient of 6.59 x 10^3^ M^-1^ [9]. Enzyme concentrations were 0.044 μM for TK0430 or 0.056 μM for AF1811. FAICAR concentrations were varied from 2–20 μM for AF1811 and from 1–8 μM for TK0430. Experimental limitations of the assay prevented data collection at concentrations lower than 1 μM, similar to previous reports [8]. Data (S2 File) were fit using the non-linear Michaelis Menten fit in GraphPad Prism, yielding K_m_ and V_max_ values and specific errors of the mean. To facilitate comparison to previously-reported proteins, specific activity was calculated from V_max_ by converting protein molarity to mg of protein for the affinity-tagged proteins.

### Temperature studies

To determine the effect of temperature on enzyme activity, the initial rates were determined in triplicate at several temperatures, using the assay conditions described above. For TK0430, initial rates were determined with 4 μM FAICAR at 50, 60, and 70°C. The enzyme concentration was 0.022 μM. For AF1811, initial rates were determined with 10 μM FAICAR at 50, 60, 65, and 70°C, and the enzyme concentration was 0.028 μM.

### Complementation

The complementation system used was as follows: ΔpurH *E*. *coli* (JW3970, part of the Keio collection [14]), were obtained from National BioResource Project, Japan. Competent cells were produced using the Inoue method [15], followed by transformation with a modified plasmid from the ASKA collection [16] that had been modified to encode only the AICAR formyltransferase portion of full-length *E*. *coli* PurH (“pPurH1”, described in S1 File). Cells containing pPurH1 were again made competent by the Inoue method and transformed with a plasmid encoding the suspected IMP cyclohydrolase gene (pAf1811 or pTk0430). Cells were grown on M9 glucose plates, supplemented with appropriate antibiotics and IMP or IPTG, as appropriate. Plates were incubated at 37°C for up to two weeks.

## Results and discussion

### Product confirmation

**Fig 2:**
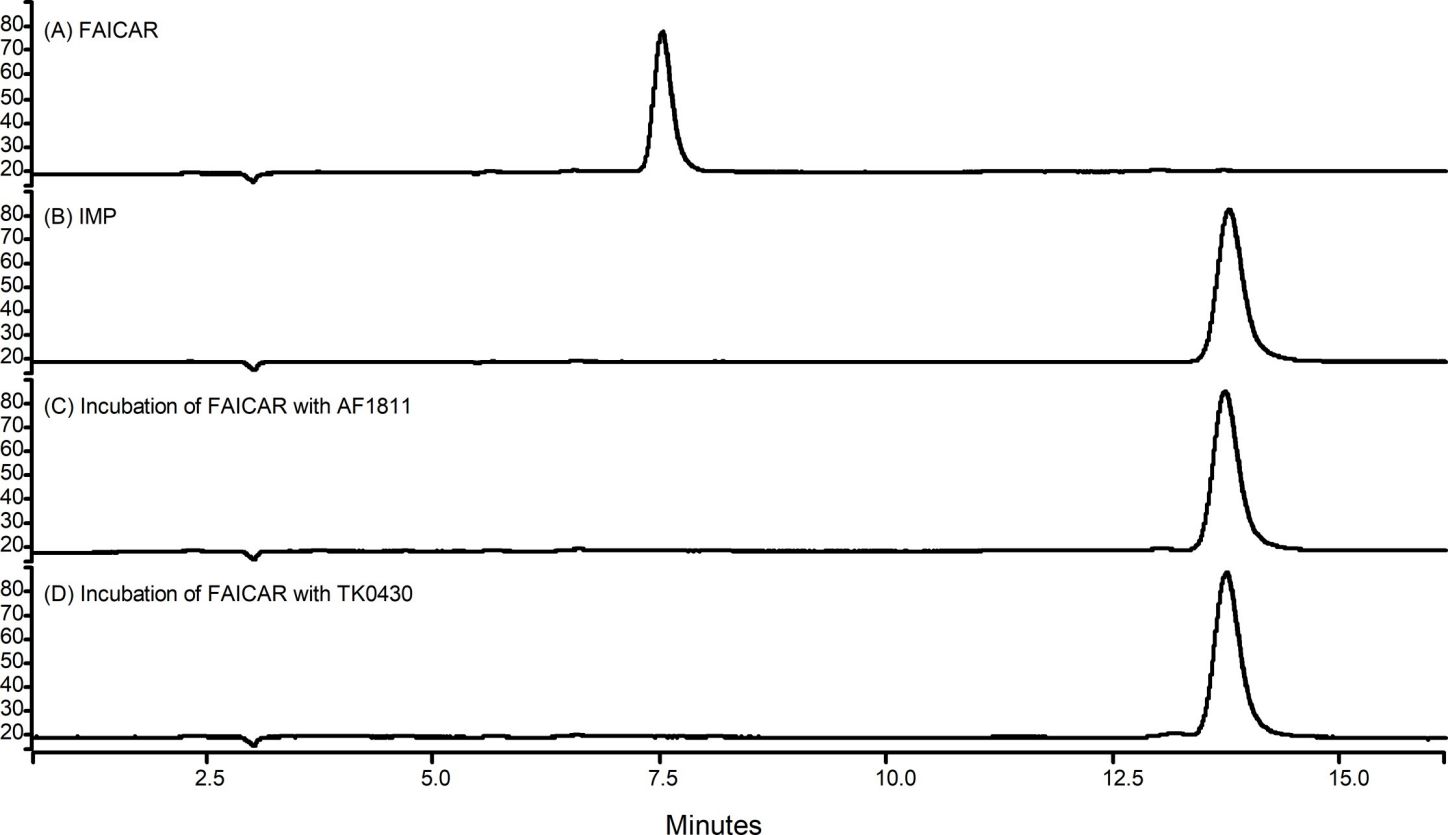
HPLC chromatograms of incubation mixtures. The Y-axis is in arbitrary units, proportional to absorbance at 248 nm. (A) Chemically-synthesized FAICAR (purified) following a 30 minute incubation at 60°C in assay buffer (Tris pH 7.3). (B) Assay buffer containing authentic IMP. (C) Assay mix as in A, with the addition of AF1811 protein. (D) Assay mix as in A, with the addition of TK0430 protein.

Synthetically-produced FAICAR incubated at 60°C with either TK0430 or AF1811 enzymes was analyzed by HPLC (Fig 2). Complete conversion of FAICAR starting material (retention time 7.5 minutes) to a product with the same retention time as an IMP standard (13.7–13.8 minutes) occurred following incubation with either enzyme, but not upon incubation with a buffer blank, showing that FAICAR does not cyclize non-enzymatically under these assay conditions. Thus, both AF1811 and TK0430 convert FAICAR to inosine-5'-monophosphate (IMP), confirming their assignments as a PurH2 and PurO (respectively).

### Complementation

Complementation assays were used to further confirm the function of the putative IMP cyclohydrolases. An *E*. *coli* strain with the *purH* gene replaced with a kanamycin resistance cassette, ΔpurH, exhibited the expected growth requirement for purines, which could be relieved by rich media or by minimal media supplemented with inosine-5'-monosphosphate. Transformation of ΔpurH with pPurH1 (encoding only the AICAR formyltransferases domain) and pMAL-c5e (the host vector used to create both pTk0430 and pAf1811) did not relieve the purine growth requirement, as no IMP cyclohydrolase activity is encoded by the PurH1 domain. However, when ΔpurH cells were transformed with pPurH1 and either pTk0430 or pAf1811, growth occurred on minimal media in the presence of IPTG, consistent with restoration of the purine biosynthetic pathway. The complementation results, summarized in Table 1, are consistent with both studied loci, TK0430 and AF1811, encoding functional IMP cyclohydrolases capable of converting FAICAR to IMP.

**Table 1:** Relative time to appearance of colonies in complementation experiments.

	Growth conditions			
Cell line	LB	M9 glucose	M9 glucose + IMP	M9 glucose + IPTG
ΔpurH	++	-	+	-
ΔpurH / pPurH1/ pMAL-c5e	++	-	+	-
ΔpurH / pPurH1/ pTk0430	++	-	+	+
ΔpurH / pPurH1/ pAf1811	++	-	+	+

All growth conditions included antibiotics for plasmid selection. (A “-” denotes no growth after two weeks. A “++” denotes wildtype-like growth, with large colonies after overnight growth. A “+” denotes 2mm colonies after 2–7 days.)

### Kinetics

**Fig 3:**
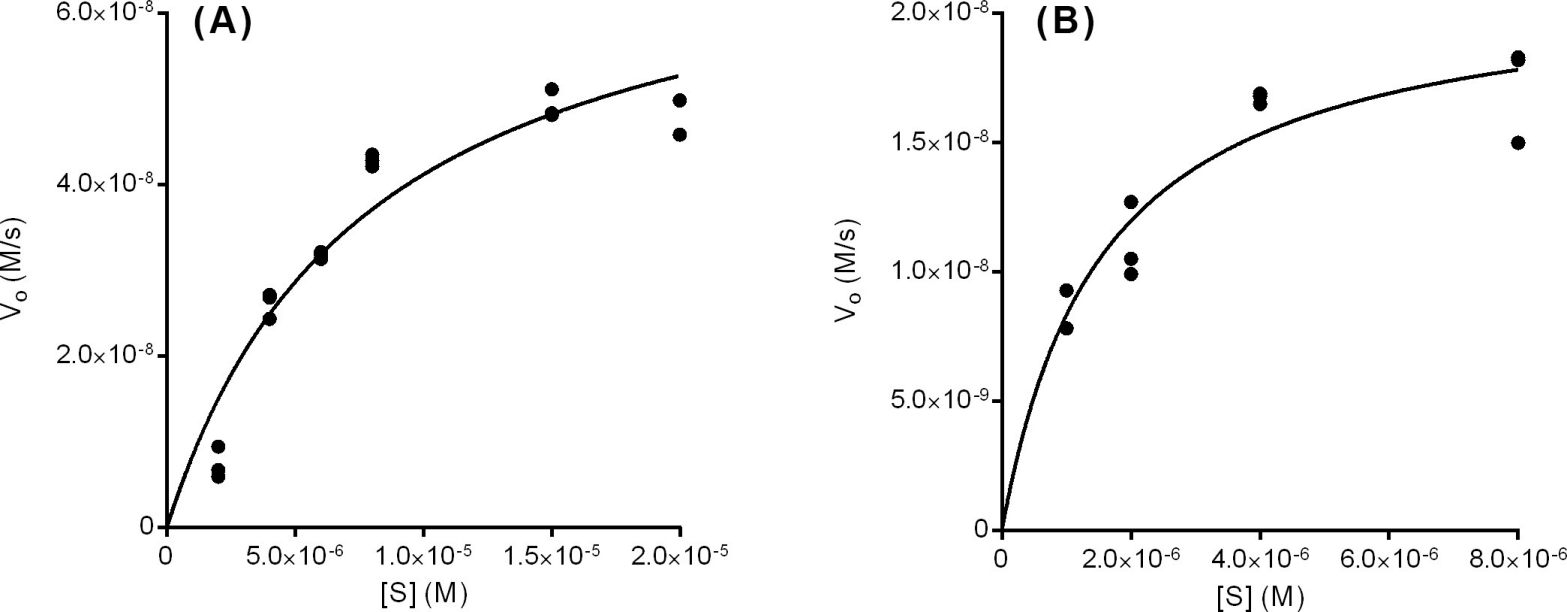
Kinetic analysis of TK0430 and AF1811. Solid lines represent best fit curves for Michaelis-Menten kinetics. (A) shows the results for the AF1811 enzyme, while (B) shows the results for the TK0430 enzyme.

Kinetics assays were performed for both enzymes, monitoring the conversion of FAICAR to IMP via absorbance change at 248nm, as previously described [9]. Initial rates were fit to the Michaelis-Menten equation (Fig 3). The K_m_ of the AF1811 protein product was estimated at 7.8 ± 1.8 μM with a k_cat_ of 1.32 ± 0.14 s^-1^ under the 50°C assay conditions. As summarized in Table 2, AF1811 has a slightly weaker binding affinity than full-length PurH enzymes from human [11] and *Staphylococcus lugdunensis* [10]. AF1811 is also less catalytically active than these two previously-assayed PurH enzymes, but this difference may be at least partially attributable to the AF1811 kinetics experiment being conducted at 50°C, well below this organism’s optimal growth temperature of 76°C [17]. Thus, the reported k_cat_ and specific activity values for AF1811 are almost certainly an underestimate of the true physiologically-relevant values.

**Table 2:** Comparison of kinetic parameters of IMP cyclohydrolase enzymes.

Enzyme	K_m_ (μM)	k_cat_ (s^-1^)	Specific activity (μmol min^-1^ mg^-1^)
*Methanocaldococcus jannaschii* PurO	82 [5]	22^a^ [5]	1.8 *[3]*
*Thermocococcus kodakarensis* PurO	1.56 ± 0.39	0.48 ± 0.04	0.45 ± 0.04
Human ATIC (PurH)	0.9 ± 0.1 [11]	8.6 ± 0.2 [11]	2.8 [9]
*Staphylococcus lugdunensis* ATIC (PurH)	2.10 ± 0.36 [10]		2.69 ± 0.56 [10]
*Staphylococcus lugdunensis* truncation (PurH2)	1.84 ± 0.29 [10]		1.75 ± 0.43 [10]
*Archaeoglobus fulgidus* (PurH2)	7.8 ± 1.8	1.32 ± 0.14	1.30 ± 0.14

Shaded rows indicate the results of this work, including standard errors of the mean. Values in unshaded rows are reproduced from the sources cited.

^a^ calculated from reported K_m_ and k_cat_/K_m_

The K_m_ for TK0430 was estimated to be 1.56 ± 0.39 μM, with a k_cat_ of 0.48 ± 0.04 s^-1^. Our assay at 50°C likely underestimates the true k_cat_ and specific activity, given that this organism grows optimally at 85°C [18]. As summarized in Table 2, the TK0430 K_m_ is 50-fold lower than the 82 μM K_m_ value reported by Graupner *et al*. [5] for the related *Methanocaldococcus jannaschii* PurO enzyme. The reported k_cat_/K_m_ ratios and specific activity for *Methanocaldococcus jannaschii* PurO [3, 5] and TK0430 PurO are more similar. The K_m_ for TK0430 is much closer to known PurH2 values than to the value previously reported for the *Methanocaldococcus jannaschii* PurO, showing that PurO-type IMP cyclohydrolases can have comparable substrate binding ability to PurH, and that weaker binding affinity for FAICAR is not a universal trait of PurO enzymes.

### Temperature effects

**Fig 4:**
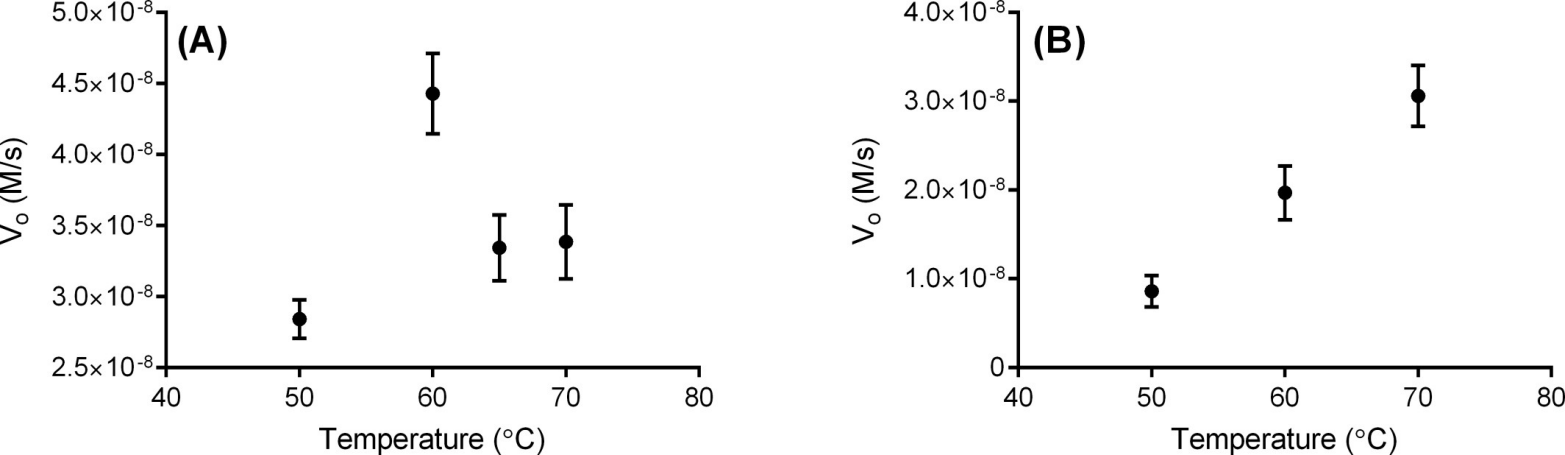
Temperature effects on TK0430 and AF1811. Each point is an average of three trials, with standard deviations. (A) shows the results for the AF1811 enzyme, while (B) shows the results for the TK0430 enzyme.

The effect of temperature on enzyme activity was assessed for each enzyme, although full kinetic characterization was not attempted at higher temperatures due to experimental limitations. As shown in Fig 4, AF1811 exhibited higher activity at 60°C than 50°C, but activity was not as high at 65°C or 70°C, suggesting an optimum temperature around 60°C for this enzyme construct under these assay conditions. AF1811 may have a higher temperature optimum *in vivo*, given that the organism’s optimal growth temperature is higher than this apparent optimum, or the activity that remains at the organism’s growth temperature may be sufficient to the organism’s needs and not under much selective pressure. TK0430 showed increased activity as temperature was increased from 50°C up to 70°C, consistent with an optimum temperature of at least 70°C.

## Conclusions

*Archaeoglobus fulgidus* AF1811 (PurH2) is a functional IMP cyclohydrolase, the first PurH2-type IMP cyclohydrolase in archaea to be experimentally confirmed, increasing the confidence of the assignments made for other archaeal genomes. Experimental characterization of a full-length PurH and an unfused PurH1 would further enhance our confidence that these gene assignments are correct but is beyond the scope of the present work.

*Thermococcus kodakarensis* TK0430 (PurO) encodes a fully functional IMP cyclohydrolase, and it is reasonable to infer that the other *purO*-like genes in the Thermococcales also encode this activity. However, the question of IMP cyclohydrolase activity in the other Thermococcales remains unsolved. The presence of an otherwise complete purine biosynthesis pathway would imply the existence of some IMP cyclohydrolase-encoding gene, but this gene remains identified. Similarly, no IMP cyclohydrolase has been identified in any of the Crenarchaeota, despite otherwise complete pathways. Although the work of White and coworkers [5, 19] on archaeal-specific AICAR formyltransferase and IMP cyclohydrolase enzymes has led to complete or near-complete proposed pathways in most Euryarchaea (except the subset of Thermococcales discussed above), additional work is needed to complete the pathway for the Crenarchaea.

## Supporting information

S1 File Plasmids and primers. This file contains a description of the plasmids constructed for this work, including primer sequences used for gene amplification.
(DOCX)

S2 File Raw kinetics data. Data used to generate Fig 3.
(XLSX)
