# Supplementary material for: Experimental characterization of two archaeal inosine 5'-monophosphate cyclohydrolases
Source: PLoS One. 2019 Oct 17;14(10):e0223983. doi: 10.1371/journal.pone.0223983 (PMC6797443; doi:10.1371/journal.pone.0223983)
Supplement: S1 File — This file contains a description of the plasmids constructed for this work, including primer sequences used for gene amplification. (DOCX) [file pone.0223983.s001.docx]

S1 File. Plasmids and Primers

All enzymes were purchased from New England Biolabs (Ipswich, MA). All PCR reactions used Phusion polymerase in HF buffer, following the package recommendations.

pPurH1 is derived from the ASKA purH plasmid, following site directed mutagenesis with the primer CC CAA CAA CGT CGT CCA GTC CGC GTG GTT GTT AAC CTG TAT CCG TTC GCC and its reverse complement. Template was digested with DpnI prior to transformation of XL1-blue competent cells. The resulting plasmid has a deletion of amino acids 8 through 100 in the purH open reading frame, comprising the majority of the predicted IMP cyclohydrolase domain in *E. coli* PurH. The final pPurH1 retains the T5-lac promoter and is IPTG-inducible. The plasmid also provides chloramphenicol resistance and the lac repressor.

pAf1811 was produced using standard directional cloning methods. The forward primer was GGGAAATTCCATATGAAGGTTCTGATTTCGTCAAGCGTTAAG and the reverse primer was GCCGAATTCTCAAGGTGCTTCATTCTCCAACCA. Purified PCR product and pMAL-c5e plasmid were both treated with the restriction enzymes NdeI and EcoRI. Double-cut vector was treated with Antarctic phosphatase. Ligations were performed using T4 DNA ligase. The resulting pAf1811 has an N-terminal maltose binding protein fused to the predicted Af1811 open reading frame. The fusion gene is preceded by an IPTG-inducible *tac* promoter. The vector additionally encodes ampicillin resistance and the lac repressor. (The full pMal-c5e vector sequence is available at <http://www.neb.com/nebecomm/tech_reference/restriction_enzymes/sequences/GenBank/pmal-c5e.gbk.txt>.)

pTk0430 was produced using the same methods as pAf1811, with the following exceptions: The primers were GGGAATTCCATATGAGGTACGTGGGAAGGACGCTC and CCTTAGCCTGCAGGTCAGAGAGAAGGAGTGAAAGAAAGCTC. The restriction enzymes were NdeI and SbfI. The pTk0430 plasmid construct has the same features as pAf1811.
